# Steps Towards Industrialization of Cu–III–VI_2_Thin‐Film Solar Cells:Linking Materials/Device Designs to Process Design For Non‐stoichiometric Photovoltaic Materials

**DOI:** 10.1002/advs.201500196

**Published:** 2016-05-17

**Authors:** Huey‐Liang Hwang, Hsueh‐Hsin Chang, Poonam Sharma, Arya Jagadhamma Letha, Lexi Shao, Yafei Zhang, Bae‐Heng Tseng

**Affiliations:** ^1^ Key Laboratory for Thin Film and Micro‐Fabrication Technology of the Ministry of EducationSchool of ElectronicsInformation and Electrical Engineeringand Photovoltaic Research CenterShanghai Jiao Tong University800 Dongchuan RoadShanghai200240China; ^2^ Institute of Electronic EngineeringNational Tsing Hua UniversityHsinchu300Taiwan ROC; ^3^ School of Physics Science and TechnologyLingnan Normal UniversityZhanjiang534048China; ^4^ Department of Materials and Optoelectronic ScienceNational Sun Yat‐Sen UniversityNo.70 Lien‐Hai RdKaohsiung80424Taiwan ROC

**Keywords:** Cu–III–VI_2_, device design, material design, non‐stoichiometric, process design

## Abstract

The concept of in‐line sputtering and selenization become industrial standard for Cu–III–VI_2_ solar cell fabrication, but still it's very difficult to control and predict the optical and electrical parameters, which are closely related to the chemical composition distribution of the thin film. The present review article addresses onto the material design, device design and process design using parameters closely related to the chemical compositions. Its variation leads to change in the Poisson equation, current equation, and continuity equation governing the device design. To make the device design much realistic and meaningful, we need to build a model that relates the opto‐electrical properties to the chemical composition. The material parameters as well as device structural parameters are loaded into the process simulation to give a complete set of process control parameters. The neutral defect concentrations of non‐stoichiometric CuMSe_2_ (M = In and Ga) have been calculated under the specific atomic chemical potential conditions using this methodology. The optical and electrical properties have also been investigated for the development of a full‐function analytical solar cell simulator. The future prospects regarding the development of copper–indium–gallium–selenide thin film solar cells have also been discussed.

## 1 Introduction

During the past few decades, the photovoltaic (PV) market has grown at a remarkable rate, particularly in thin film solar cells (TFSCs) which are on their way to become one of the major sources of electricity production around the whole globe. It was predicted that the market share for Cu–III–VI_2_ TFSCs will be 5% by year 2010 and 15–20% by year 2020. However, research and development of copper–indium–gallium–selenide TFSCs are still in a crucial phase due to fundamental obstacles—such as low production yields, non‐reproducibility, and non‐uniformity over large area—confronted during the industrialization and commercialization of CIGS thin film solar cells. Concerning such issues, we published our first research paper in year 2003.^1^ After this, the concept of in‐line sputtering and selenization has become the international standard globally. In the last decades, dozens of CIGS manufacturing units have been established worldwide, but claims of successful production are rare, due to difficulty in predicting and controlling the local chemical composition distribution of the film, despite the availability of commercial device simulation tools. Moreover, the production efficiency of large‐area PV cells and panels varies in a wide range from 6% to 13%.

In 2003, we again published research^2^ pointing out the problems encountered during the commercialization. Over the years, many experimental and theoretical research works published in many journals have focused on various subjects of problems and its solutions for CIGS TFSCs.^3^, ^4^, ^5^, ^6^, ^7^, ^8^, ^9^, ^10^

**Figure 1:**
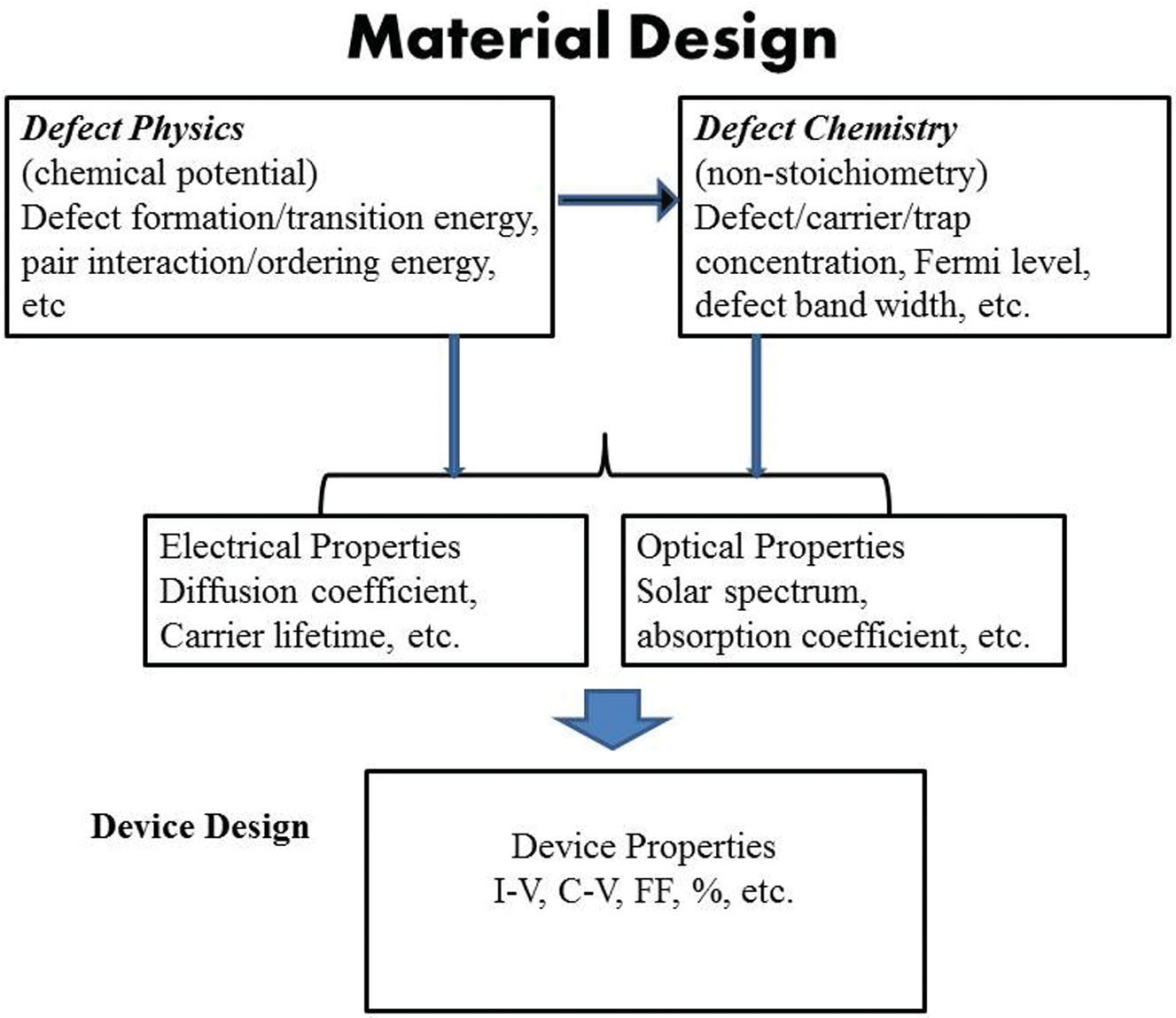
The scheme of our materials design and device design in which the electrical and device properties are optimized, and related to the process parameters such as chemical potential and non‐stoichiometry. Reproduced with permission.^2^ Copyright 2003, Elsevier.

In 2013, we published one more research article.^11^ This article pointed out how the new concept of metal organic sputtering could be used for the fine tuning and tailoring of film compositions based on the programmed material and device design. The scheme of our intelligent material and device design, shown in **Figure** **1**, described the detailed calculation of the neutral defect concentrations of non‐stoichiometric CuInSe_2_, CuGaSe_2_ and ZnO under specific atomic chemical potential conditions (*μ*_x_ = 0, X = Cu, In/Ga, Zn).

These calculations are the main procedure of the intelligent design and key to the device and process design. The carrier concentration and the electrical properties of these materials with variable atomic constitutions are further calculated which demonstrate the main functions of the computer aided design (CAD) tool used.^2^, ^12^, ^13^, ^14^

In the present Review, we sum up these designs to get in‐depth knowledge about the chemical compositional variation to obtain the complete set of process control parameters. These design tools will help moderately to overcome all the obstacles encountered during industrialization of CIGS thin film solar cells. The future aspects for industrialization of CIGS TFSCs are also discussed.

## 2 Brief Discussion of Intelligent Material Design

### 2.1 Experimental Data

**Figure 2:**
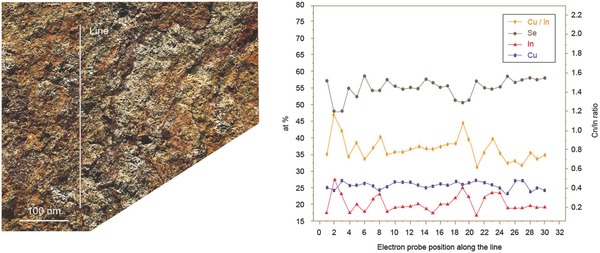
Chemical composition of a near‐stoichiometric CuInSe_2_ film measured consecutively by using an electron beam with a 3 nm probe size along a line marked on a TEM image (left); the data was plotted (right) and summarized in Table 1.

It's well known that Cu–III–VI_2_ typically have a wide phase stability region, which extends a few atomic percent of the chemical composition of the thin film, in contrast to III–V and II–VI compounds. The chemical composition for a near‐stoichiometric CuInSe_2_ is shown in **Figure** **2**. The variations in CuInSe_2_ film were detected using a transmission electron microscope (TEM) equipped with a field emission gun.^11^ A similar result had also been reported for CIGS thin film.^12^

**Table 1:** Chemical composition of near‐stoichiometric CuInSe_2_ films shown in Figure 2, 11

	Cu [at %]	In [at %]	Se [at %]	Cu/In
Maximum	28.2	28.1	57.1	1.18
Minimum	17.2	22.6	47.9	0.62
Average	20.9	25.9	53.3	0.81
Standard Deviation	2.73	1.37	2.80	0.12

### 2.2 Theoretical Data

In 2003, we published an example of the design tool using the concept of minimization of total free energy^2^, ^13^, ^14^ which includes the configurational entropy, defined as;

$$G\left(T,\mathrm{Crystal size}\right)=\Sigma n_{i}E_{\mathrm{fi}}-TS_{\mathrm{config}},$$

(1) $$S_{\mathrm{config}}=\;K\;\ln\frac{N_{\mathrm{total}}!}{\pi_{j}N_{j}!}$$

Where *n_i_* is the number of the *i*th defect, *E*_fi_ is the formation energy, *T* is the temperature, *S*_config_ is the configurational entropy, *K* is the Boltzmann constant, *N*_total_ is the total number of lattice sites, *N_i_* is the total number of defect sites of the *i*th defect. We find the possible results for the co‐existence of donors and acceptors in CuMSe_2_ (M = In, Ga), which includes either the new defects produced through interaction or donor–acceptor pair/cluster formation.

**Table** **2** shows the defect formation energies and the defect transition levels. We also find the possible phases of CuMSe_2_ resulted due to the compensated donor–acceptor pairs in different Cu concentration, for example Cu_1_M_3_Se_5_ phase is observed by 80% Cu_1_M_5_Se_8_ and 20% CuMSe_2_.^19^

**Table 2:** The defect formation energies and defect transition levels used in our calculations^19^

CuInSe_2_		E_for_	E_D_/E_A_	CuGaSe_2_		E_for_	E_D_/E_A_	ZnO		E_for_	E_D_/E_A_
V_Cu_	0	0.60	E_v_ + 0.03	V_Cu_	0	0.66	E_v_ + 0.01	V_Zn_	0	10.6	E_v_ – 0.5
	–1	0.63			–1	0.67			–1	10.1	E_v_ + 0.0
									–2	10.1	(E_v_ – 0.25)
V_In_	0	30.4	E_v_ + 0.17	V_Ga_	0	2.83	E_v_ + 0.19	V_O_	+2	–3.0	E_c_ + 1.2
	–1	3.21	E_v_ + 0.41		–1	3.02	E_v_ + 0.38		+1	1.5	E_c_ – 2.4
	–2	3.62	E_v_ + 0.67		–2	3.40	E_v_ + 0.66		0	2.4	(E_c_ – 0.6)
	–3	4.29			–3	4.06					
V_Se_	+2	1.12	E_c_ – 0.1	V_Se_	+2	1.01	E_c_ – 0.38	Zn*_i_*	+2	–2.3	E_c_ + 1.1
	+1	–			+1	–			+1	2.1	E_c_ + 0.8
	0	3.00			0	3.61			0	6.2	(E_c_ + 0.95)
Cu*_i_*	+1	2.04	E_c_ – 0.20	Cu*_i_*	+1	1.91	E_c_ – 0.21	O*_i_*	0	9.7	E_v_ + 0.7
	0	2.88			0	3.38			–1	10.4	E_v_ + 1.7
									–2	12.1	E_v_ + 1.2
Cu_In_	0	1.54	E_v_ + 0.29	Cu_Ga_	0	1.41	E_v_ + 0.29	Zn_O_	+2	0.4	E_c_ + 1.5
	–1	1.83	E_v_ + 0.58		–1	1.70	E_v_ + 0.63		+1	5.2	E_c_ + 1.1
	–2	2.41			–2	2.33			0	9.6	(E_c_ + 1.3)
In_Cu_	+2	1.85	E_c_ – 0.34	Ga_Cu_	+2	2.04	E_c_ – 0.69				
	+1	2.55	E_c_ – 0.25		+1	3.03	E_c_ – 0.49				
	0	3.34			0	4.22					
V_Se_ + 2V_Cu_	0	3.63	–	V_Se_ + 2V_Cu_	0	3.65	–				
V_Se_ + Cu_In_	0	–	–	V_Se_ + Cu_Ga_	0	–	–				
2Cu*_i_* + Cu_In_	0	3.11	–	2Cu*_i_* + Cu_Ga_	0	3.20	–				
In_Cu_ + 2V_Cu_	0	0.33	–	Ga_Cu_ + 2V_Cu_	0	0.74	–				

**Figure 3:**
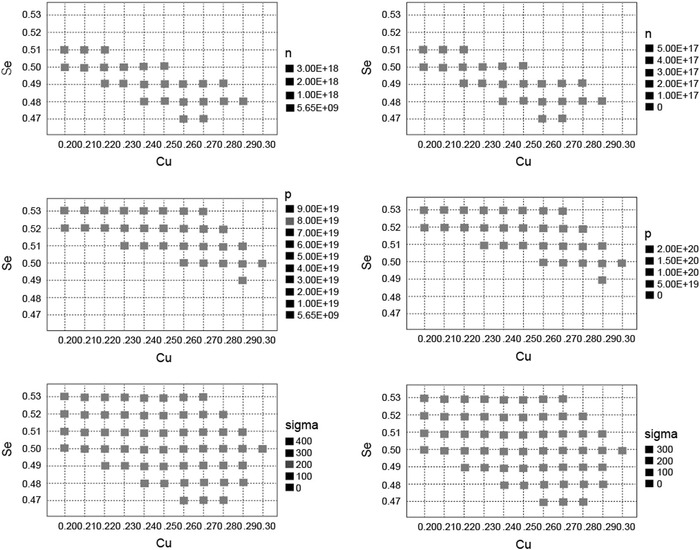
The calculated carrier concentration and electrical conductivity of CuInSe_2_ (left) and CuGaSe_2_ (right) at 300 K.

**Figure 4:**
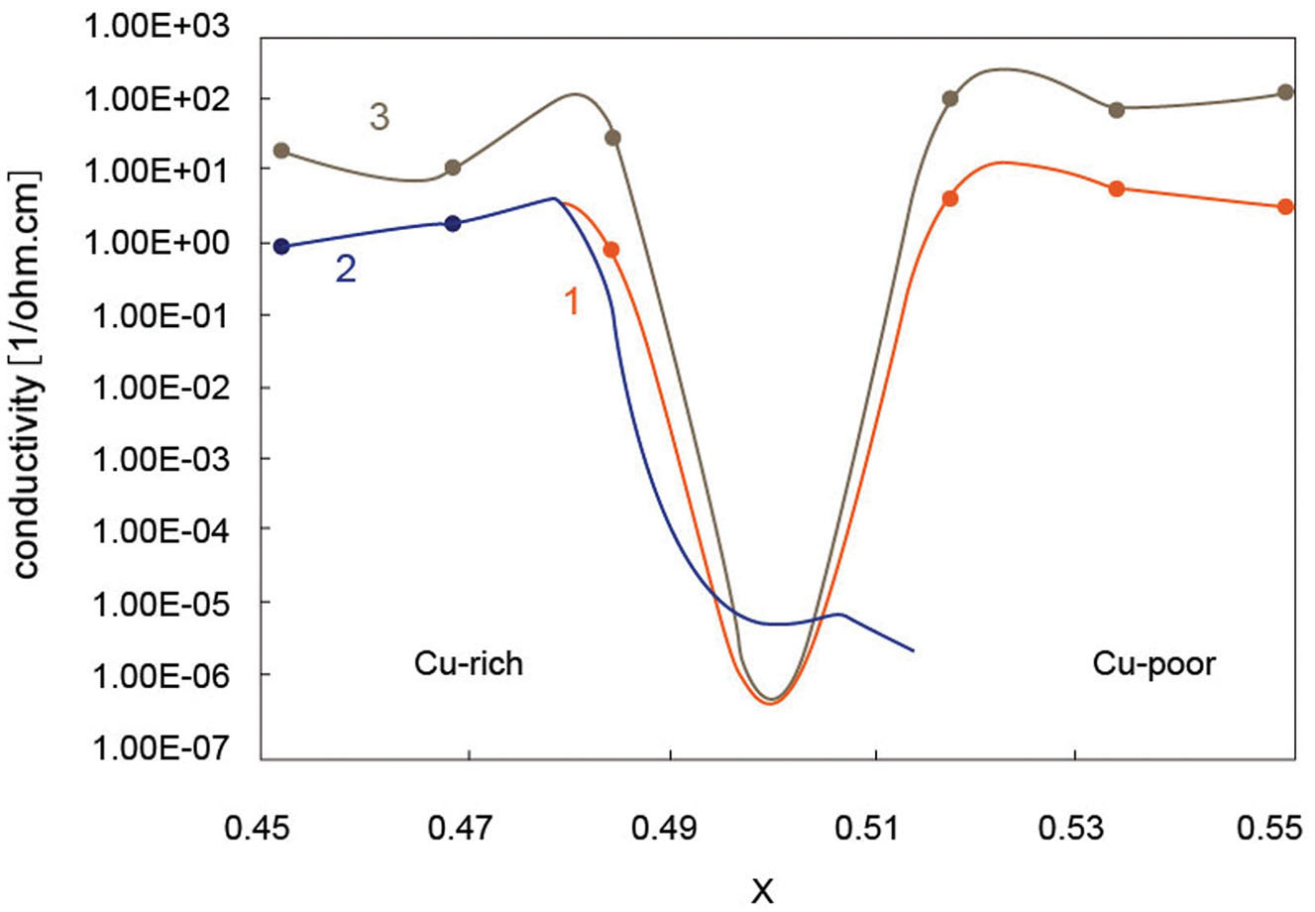
The measured conductivity of the CuInSe_2_ thin film along the Cu_2_Se‐ In_2_Se_3_on‐tie line with composition (Cu_2_Se)_1–_ *_x_*(In_2_Se_3_)*_x_* (line 1) and the calculated range (between line 2 and 3) near the Cu_2_Se–In_2_Se_3_ tie line at 300 K.

For solving the charge neutrality equation a few parameters are required, such as the carrier concentrations, ionized/neutral defect concentrations, and Femi level at a certain temperature. **Figure** **3** shows the calculated carrier concentration and electrical conductivity of CuInSe_2_ and CuGaSe_2_ at 300 K. Note that in **Figure** **4** **,** for the film composition from stoichiometry to Cu‐poor, the conductivity will rise first and then fall down, the conductivity is even lower than that of the stoichiometry.

## 3 Brief Discussion of Device Design

For numerical analysis of CIGS solar cells, the device simulator SCADS 3.2^15^ has been widely used. In 2014, Naoki Ashida et a1.^16^ simulated the 19% efficiency of a solar cell in which the 2 μm thick CIGS absorber layer was divided into two regions, such as low defect density layer (front side) and high defect density layer (back side). The thickness of low defect density layer resulted in 97% of cell efficiency on assuming a fixed bandgap of 1.2 eV and a carrier diffusion length of 0.72 μm.

We also developed an alternate full‐function (indoor, outdoor, *I–V*, *C–V*) analytic solar cell simulator, in which the following (time‐independent) device equations are considered.

a. The continuity equation

(2) $$0\;=\;-\frac{1}{q}\frac{d}{dx}\tau_{p}+G_{p}-R_{p},\;R_{p}=\frac{\Delta p}{\tau_{h}},\;\Delta p\;=p_{n}\;-p_{n0}$$

(3) $$0\;=-\frac{1}{q}\frac{d}{dx}\tau_{n}+G_{n}-R_{n},\;R_{n}=\frac{\Delta n}{\tau_{e}},\;\Delta n\;=n_{p}\;-n_{p0}$$

b. Transport equations

(4) $$\tau_{p}=\;pq\mu_{p}\;E-qD_{p}\frac{dp}{dx}$$

(5) $$\tau_{n}=\;nq\mu_{n}\;E+qD_{n}\frac{dn}{dx}$$

**Figure 5:**
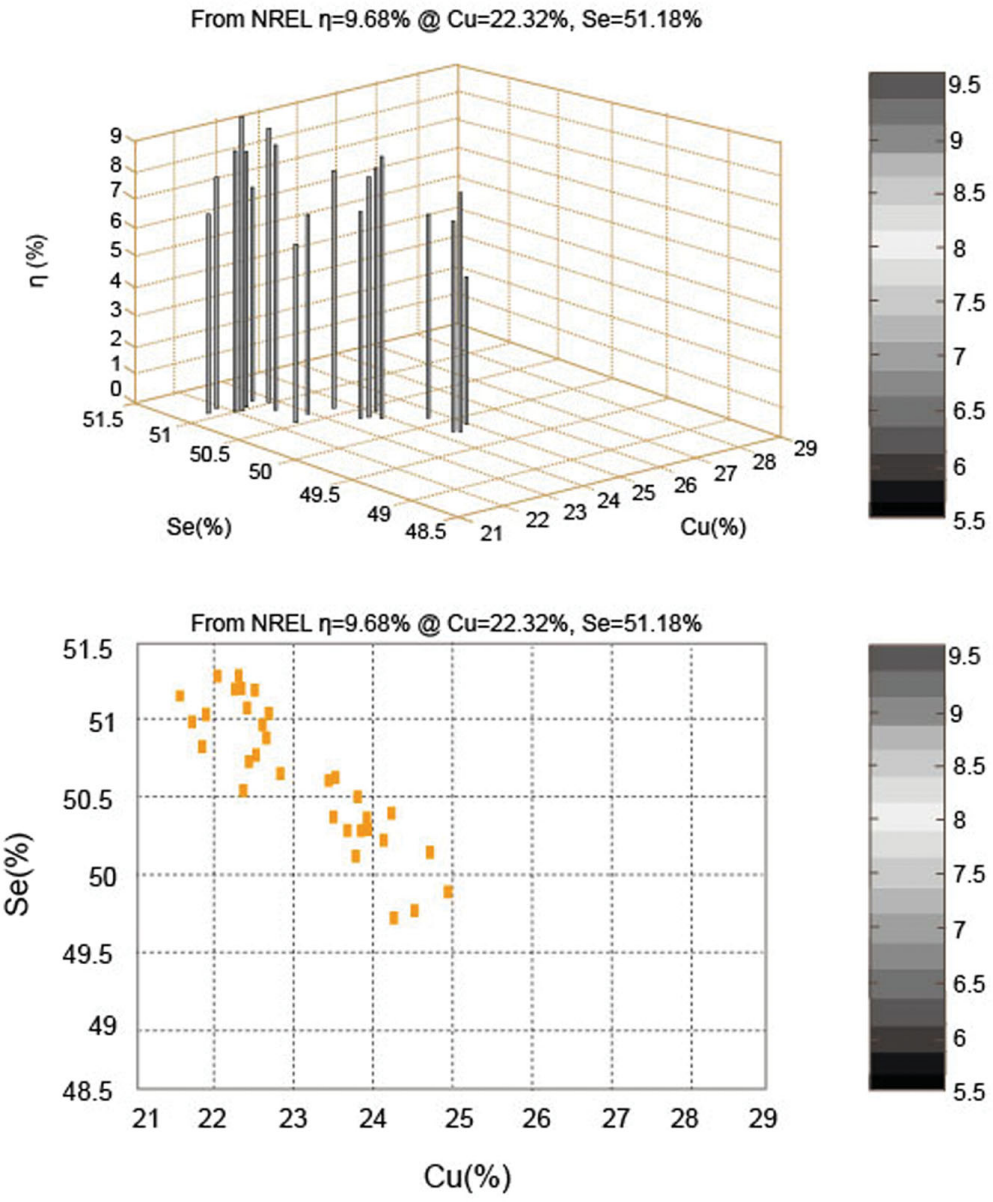
The comparison of the computed efficiencies as‐compared with NREL's database of ZnO/CdS/CuInSe_2_ solar cells: (upper) 3D view, (lower) 2D view. Reproduced with permission.^43^

All the boundary conditions (front contact, n‐QNR/SCR interface, p‐QNR/SCR interface, and back contact) are considered, where QNR stands for quasi neutral region and SCR stands for space charge region for a typical n‐CdS/p‐CIGS device structure. The example of our simulation results show that the higher efficiency cells are distributed along the line from Cu:Se = 0.3:0.5 to the stoichiometric point and the line from Cu:Se = 0.21:0.52 to the stoichiometric point. For comparison of the computed efficiencies with the NREL experimental data, the consistence are shown in **Figure** **5**. The device structures of these cells are ZnO/CdS/CuInSe_2_. The atomic compositions of the higher efficiency cells are concentrated near Cu:Se = 0.22:0.51 or along the line from Cu:Se = 0.22:0.51 to the stoichiometric point.

**Figure 6:**
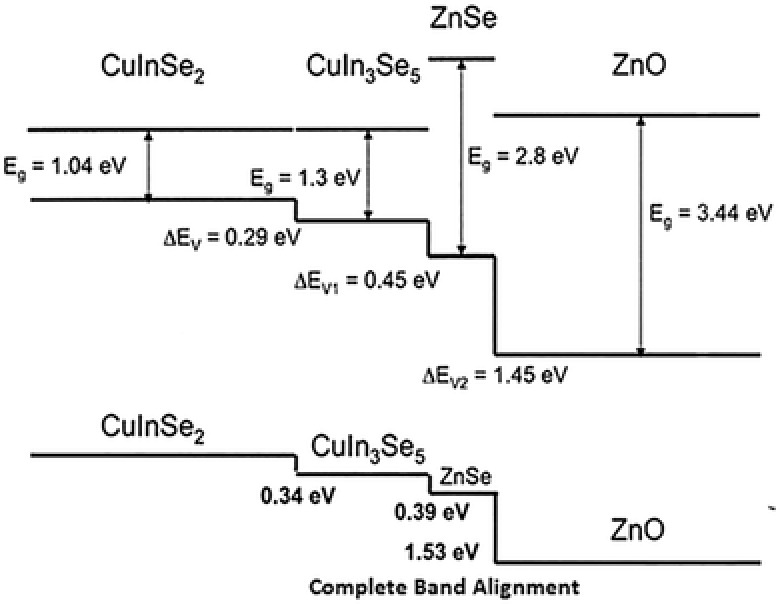
The complete band alignment includes the copper depletion at the interface. Reproduced with permission^19^ Copyright 1998, American Institute of Physics.

**Figure 7:**
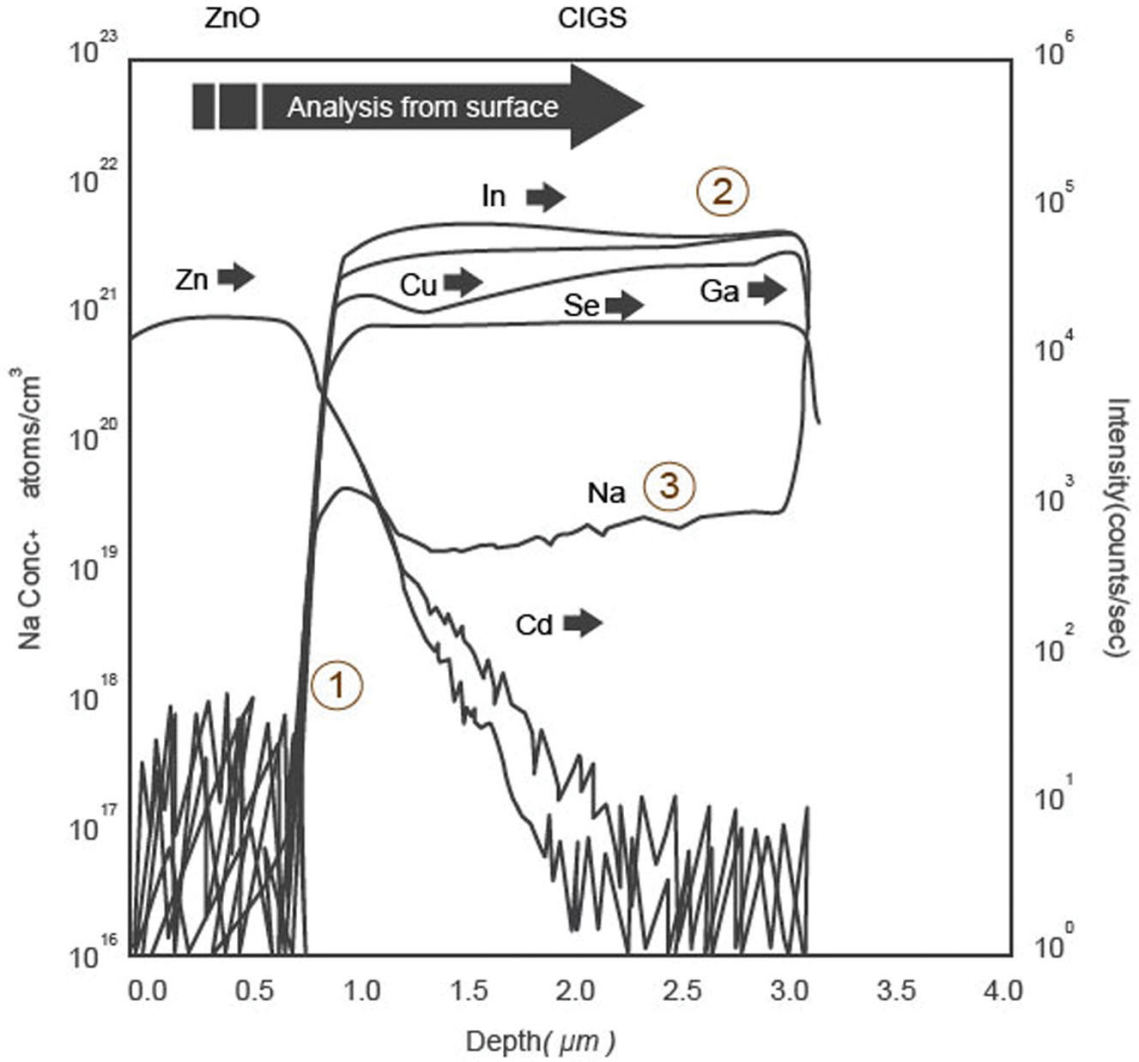
SIMS depth profile of Cu, In, Ga, Se, Cd and Na. 1) CdS/CIGS and ZnO diffusion. 2) Cu, In, Ga, and Se depth profile. 3) Quantitative analysis of Na in CIGS.^20^

In the last decades, many studies on interface and surface compositional profile have dealt with the advanced characterizations for the high efficiency CIGS solar cells. A few examples are:

1. Conduction band profiles are changed by the three stage selenization.^17^
2. Ordered defect compounds (ODC: CuInSe_2_, CuIn_3_Se_5_, CuIn_5_Se_8_ etc.) obtained from CuInSe_2_/CIGS solar cell studies revealed the depletion of Cu near the surface in X‐ray photoelectron spectroscopy (XPS) investigations, ordered defect compound structured was predicted by theory as well experiments.^18^
3. Low energy electron diffraction (LEED), angular‐resolved ultraviolet photoelectron spectroscopy (ARUPS), and auger electron spectroscopy (AES) investigations show, in **Figure** **6**, that the CuInSe_2_ surface is stabilized by defect pairs (2V_cu_ and In_cu_) and band alignment gives hole barrier at the interface.^19^
4. Secondary ion mass spectrometry (SIMS) depth profile of Cu, In, Ga, Se,Cd and Na revealed the CdS/CIGS/ZnO diffusion phenomena, as shown in **Figure** **7**.^20^
5. Defect in grains and grain boundaries.^21^

The knowledge of electronic properties of the interfaces in semiconductor devices is critically dependent on the detailed atomic structure of the contact plane. Thus, the attempt to model the junction in chalcopyrite thin films by well‐defined interface to classify the influence of grain boundaries, lateral inhomogeneity and chemical variations in compositions and their distributions across and aside the contact planes. All this information can be obtained utilizing modern analytic tools, like XPS, ultra‐violet photoelectron spectroscopy (UPS), LEED, scanning tunneling microscopy (STM), and X‐ray photoemission electron microscope (XPEEM). In situ band alignment, band broadening, chemical reacted interfaces, and crystalline structure with high resolution have been determined by experiments and incorporated with our material analysis for better accuracy and reproducibility obtained in the device design for the future industrial applications.

## 4 Brief Discussion of Process Design

In previous sections, the two aspects including structure and composition greatly affect the opto‐electrical properties of the polycrystalline semiconductors. In this section, we describe better understanding of the way to generate poly structure through careful observation of the particle transfer procedures during the manufacturing processes after knowing the final positions of the deposited atoms and the materials microstructures. On controlling and modifying the process, the composition as well structure can be tuned directly by varying the process parameters to acquire the desired opto‐electrical properties.

The original Thornton's zone model^23^ describes the metallic grain structures according to the sputtering gas pressure and the substrate temperature. The magnetron sputtering process is most preferred for the industrial application since the deposition rate is very high. Also, the energy dependent sputter yield is noted. Ellmer^24^ further built a model to illustrate the inter‐relationships between the process parameters (like substrate temperature and deposition rate) and the structural/opto‐electrical properties. However, the pressure (particles momentum) effect is not considered in this model.

### 4.1 Berg's Model

This model is particularly used for the ZnO reactive sputtering. Basically, the surface coverage on the deposited film is just the composition of the film, but the composition is not easy to control since the system is unstable and it requires the plasma diagnose sensor to undergo feedback control. The basic idea of Berg's model,^25^, ^26^ where the changes of the number of absorbed oxygen atoms per unit area *N_x_* at the surfaces of the target is:

(6) $$\frac{dN_{T}}{dt}=\;2\alpha_{t}\left(1-\theta_{1}\right)-J/eS_{N}\theta_{1}$$

at the substrate:

(7) $$\begin{array}{c} \frac{\;dN_{S}}{dt}=\;2\alpha_{C}F\left(1-\theta_{2}\right)+\frac{J}{e}S_{N}\theta_{1}\frac{A_{t}}{A_{C}}\left(1-\theta_{2}\right)\\ \;\;-\;\;\frac{J}{e}S_{M}(1-\theta_{1})\frac{A_{t}}{A_{C}}\theta_{2} \end{array}$$

**Figure 8:**
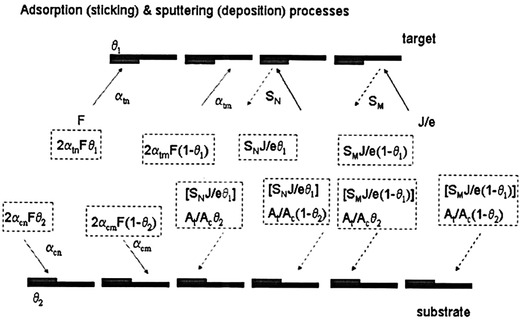
The basic idea of Berg's model. Reproduced with permission.^25^ Copyright 1981, American Institute of Physics.

Where *α_x_* are the sticking coefficients, *S*_X_ are sputtering yields, and *θ_x_* are the coverage. **Figure** **8** shows *θ* an example of the simulation result of the ZnO reactive sputtering system (*θ*_2_ always <1). We could also use Berg's model to predict the composition of the compound thin film, we can also predict if the operating point is stable. Thus, we can study the time‐dependent behavior of the reactive sputtering system.

From Berg's model,^26^ we can get expressions of *θ*_1_, *θ*_2_ and *R*, the total sputtering rate (consumption of the target) in

(8) $$R\;=\;\frac{J}{e}\left[S_{N}\theta_{1}+S_{M}\left(1-\theta_{1}\right)\right]$$

**Figure 9:**
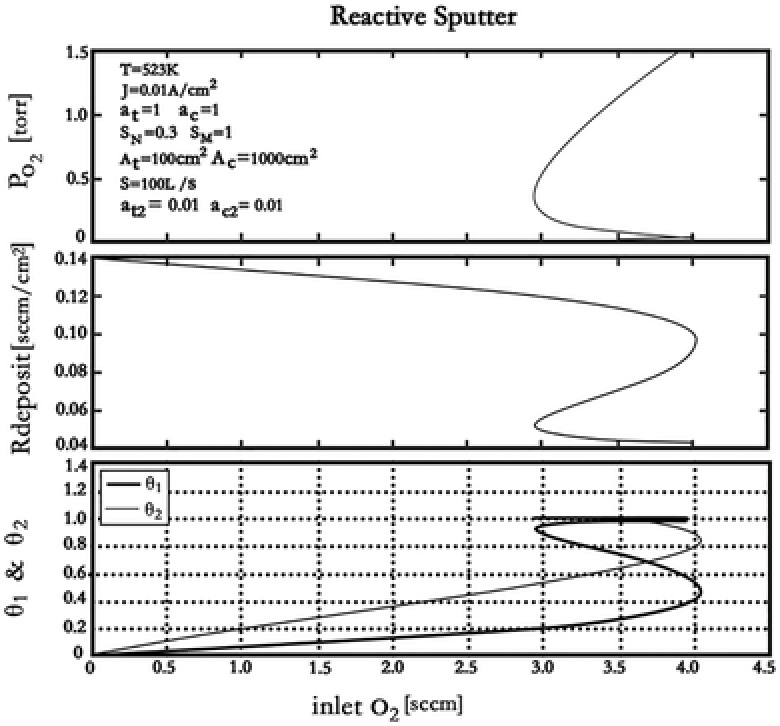
A simulation result of the ZnO reactive sputtering system.^47^

And we can generate some lines passing the origin of the Rplot, each line represents a specific *θ*_2_ value. However, our goal is to know if it is possible to control the composition by controlling *θ*_2_. **Figure** **9** shows a simulation result of the ZnO reactive sputtering system and we want to know if it is possible for *θ*_2_ to be larger than one.^47^

### 4.2 CISe RTP—IEC's Model

After we finish the CuInSe_2_ defect concentration calculations, we can apply the result to the model which was developed by IEC^27^ to predict the processing time under a certain process temperature.

**Figure 10:**
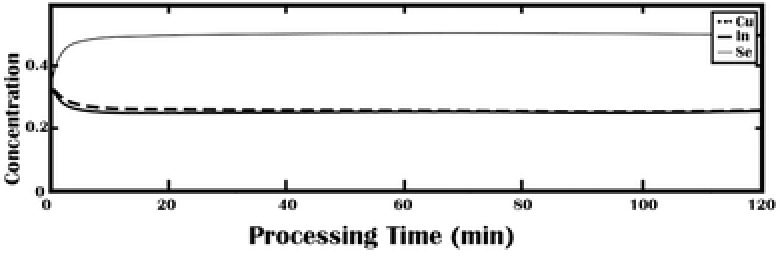
The Se or H_2_Se RTP of Cu: In:Se = 0.24:0.25:0.51 Cu*_x_*In*_y_* films at 450 °C, *μ*_Cu_ = 0, *μ*_In_ = 0; the mole ratio of the constitute atoms (in the film) as functions of the processing time (120 min) Film volume = (2.5 cm)^2^ × 2 μm).^47^

For CuIn

(9) $$2\mathrm{In}+\mathrm{Se}\to {\mathrm{In}}_{2}\mathrm{Se}......K_{2}$$

(10) $${\mathrm{In}}_{2}\mathrm{Se}+\mathrm{Se}\to 2\mathrm{InSe}\;......K_{3}$$

(11) $$2\mathrm{CuIn}+2\mathrm{Se}\to {\mathrm{Cu}}_{2}\mathrm{Se}+{\mathrm{In}}_{2}\mathrm{Se}\;......K_{a}$$

(12) $$2\mathrm{InSe}+{\mathrm{Cu}}_{2}\mathrm{Se}+\mathrm{Se}\to 2{\mathrm{CuInSe}}_{2}\;......K_{7}$$

where $\;K_{i}=K_{i0}\;\exp^{\left(-\frac{E_{ai}}{RT}\right)}$ and *V* =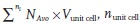, is the film volume taken as a time‐independent constant. $n_{i}=[i]\;\;\times \;V$ are the mole numbers, $n_{\mathrm{unit}\;\mathrm{cell}}$ are the number of pairs of the atoms in the unit cells. **Figure** **10** shows an example of the Se or H_2_Se RTP of Cu:In:Se = 0.24:0.25:0.51 Cu*_x_*In*_y_* films at 450 °C, *μ*_Cu_ = 0, *μ*_In_ = 0; the mole ratio of the constitute atoms (in the film) as functions of the processing time (film volume = (2.5 cm)^2^ × 2 μm). In our non‐stoichiometric case, the initial mole concentrations of Cu and In taken from our calculated data. Then, these were input to the Cu*_x_*In*_y_* and In mole concentrations in order to solve the ordinary differential equation system.

**Figure 11:**
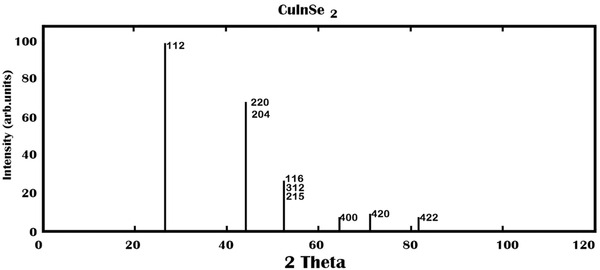
Simulated XRD spectra of non‐stoichiometric CuInSe_2_; Cu:In:Se_2_ = 0.21:0.26:0.53.^47^

Regarding building the non‐stoichiometric structures, we build the X‐ray defraction (XRD) spectrum. The supercell values have been used to calculate the XRD spectrum of non‐stoichiometric CuInSe_2_ and CuGaSe_2_. In our work, the method described by Attia et al.^28^ has been incorporated and only modify the structural factor by summing over all the atoms in the supercell. The defect site in each defect cells has been chosen randomly. We observed the presence of extra small peaks in the XRD spectrum, as shown in **Figure** **11**.^47^

nomalous neutron diffraction scattering of synchrotron X‐ray radiations gives more accurate data of composition distributions.^28^ However, in situ XRD is the most convenient tool for monitoring the deviations from the stoichiometric compositions. A substantial increase in full wavelength half maximum (FWHM) indicates an incapability gap, in which the lattice constants depends on Ga/III and follow Vegrad's law.^29^, ^30^, ^31^ More work should be done to make it more feasible to be used for industrial use for future process monitoring and control.

## 5 Some Means to Improve Film Composition Control

**Figure 12:**
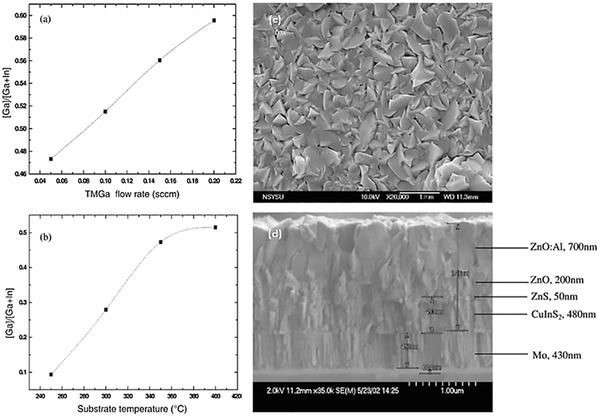
Preliminary result on the metal organic sputtering of CIGS photovoltaic. a) Ga content as a function of TMGa flow rate. b) The content of Ga as a function of substrate temperature. c) Plain view SEM image of the deposited CIGS film. d) Cross‐sectional SEM image of a CIGS photovoltaic. Reproduced with permission.^1^ Copyright 2003, Elsevier.

**Figure 13:**
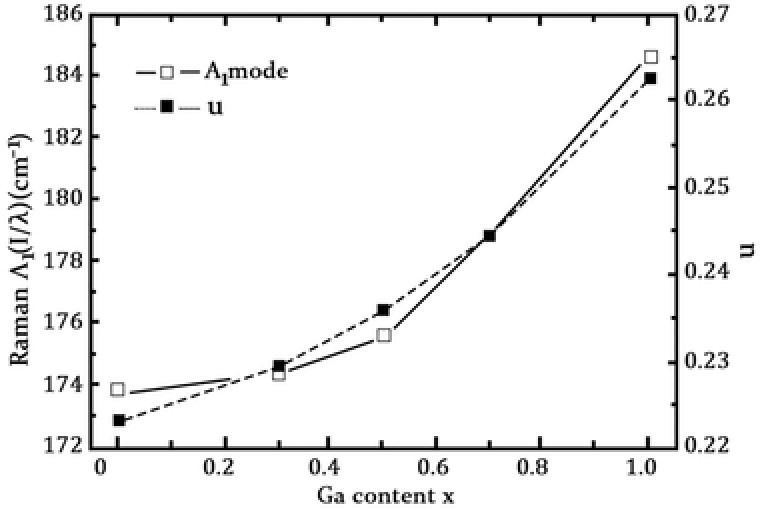
Room temperature Raman shift inCuIn_1–_ *_x_*Ga*_x_*Se_2_ thin films of thickness 600 nm deposited on glass substrate with change in Ga content, the Raman peak shifted from 173.8 cm^−1^ to 184.6 cm^−1^ in A1 mode and *u* is Se shift parameter. Reproduced with permission.^33^, ^35^

**Figure 14:**
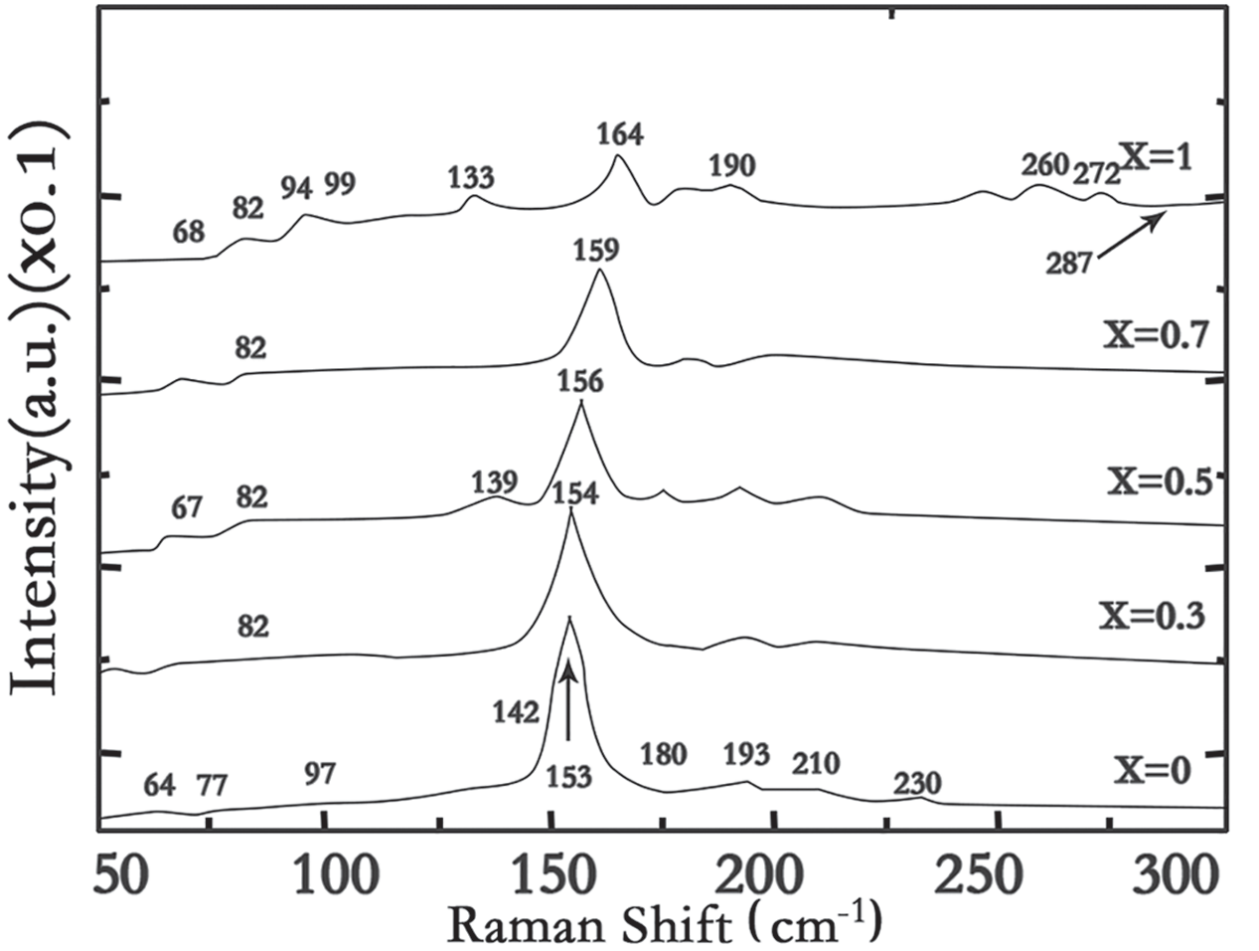
Room temperature Raman shift in Cu(In_1–_ *_x_*Ga*_x_*)_3_Se_5_ thin films, of thickness 600 nm on glass substrate, with change in Ga concentration.^34^, ^35^

During the fabrication of CIGS thin films, there will be chemical fluctuation‐induced nano‐domains. A novel metal–organic sputtering (MO‐sputtering) technique was developed in our work, in which a metal–organic compound like TMGa (trimethygallium) as the reactant was used during the reactive sputtering procedure. **Figure** **12** shows the change of Ga/(Ga + In) ratio as a function of TMGa flow rate and substrate temperature as well,^1^ and the linear relationship of Ga/(Ga + In) with the TMGa flow rate adjust the deposit thin film composition is particularly interesting. Note also in **Figure** **13** and **Figure** **14** that the result from the micro‐Raman spectroscopy, in which the Raman shift as a function of the film composition change of Cu/(Ga + In) is presented. It is clear that the micro‐Raman shift is sensitive as the composition change of the CIGS thin films. To combine the use of MO‐sputtering for the film growth and its feedback monitoring with the Raman shift, it might provide a means to better control the stoichiometry of thin film during the manufacturing steps.

From the Raman scans, we can observe that the Raman shift is sensitive to the composition change in the CIGS thin films. The continuous monitoring of CIGS thin films grown using MO‐sputtering with Raman shift provides better control on the stoichiometry of thin film during the fabrication process. This allows them to achieve a fundamental improvement of the manufacturing technology.

Recent report by Rommel Noufi of NREL, USA addressed on science and technology of high efficiency CIGS thin film solar cells,^41^, ^43^, ^44^, ^45^, ^46^ which include schematic profiles of “3‐stage process for CIGS”, fundamental understanding of defects in the bulk and surface regions,s tudies on the device structure, properties of thin film layers, growth dynamics of the CIGS films; focusing on the restructured surface, development of grain and grain boundary structures model, etc. are dealt in‐depth. Most studies are related to the film compositional profiles.

An alternate way is that once the film compositional profiles and their distributions are accurately determined from our theory and experimental analysis, that is the essential electrical and optical properties can be computed from our material design and device design, these data can be checked from reports in the literatures,^41^ and they are meaningfuld evice structural parameters to be loaded into the renowned device simulator SCADS 3.2^15^ to get the photo conversion efficiencies, and a “big database” can thus be constructed, and they should help improve the production yields for cells and modules manufacturing.

## 6 Future Prospects

Moreover, the status reports on different stages of CIGS developments have been periodically published by NREL, USA and Fraunhofer ISE Germany.^36^, ^37^, ^38^, ^39^ These reports assisted the promotion of technology development and industrialization of thin‐film CIGS produces. Although, many challenges still lie ahead, including optimization and control on the CIGS absorber film stoichiometry, interface and film uniformity over large areas for the power module fabrication.

**Figure 15:**
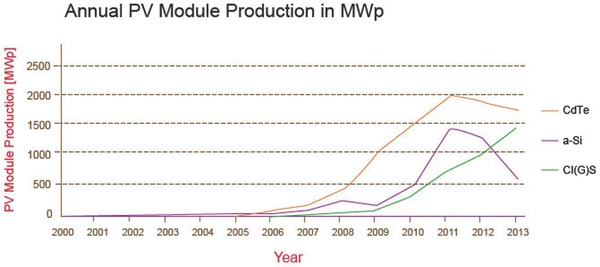
Thin‐film technologies worldwide annual PV module production in MWp.^41^

Nowadays, despite PV production of monocrystalline Si and multicrystalline Si dominating in the PV market, the importance of TFSCs will steadily rise in the coming decade; moreover, in recent time the CIGS already win its own counter parts like CdTe and a‐Si (**Figure** **15**).^40^, ^41^

**Figure 16:**
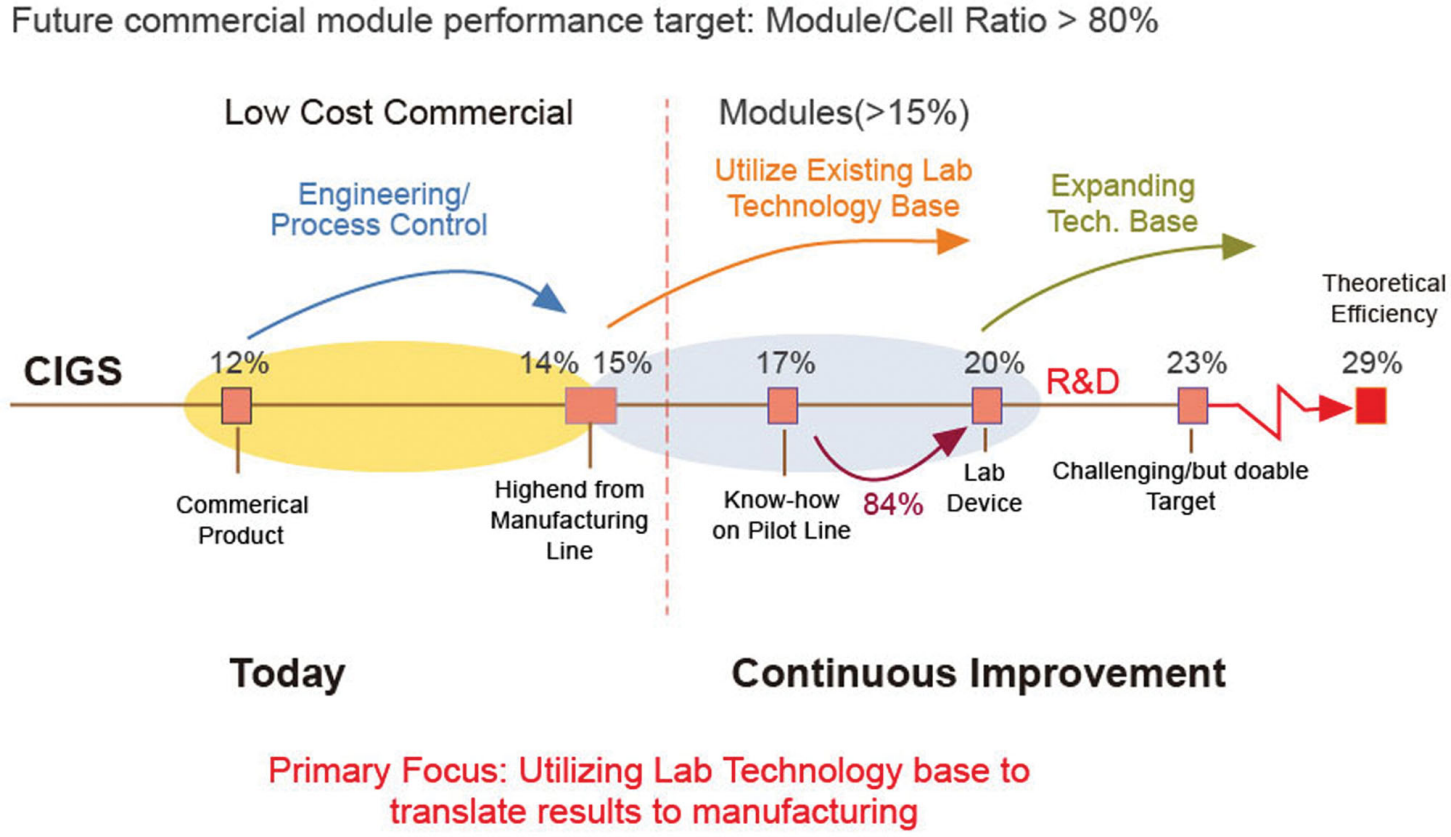
Closing the gap between laboratory cells and modules.^41^

As viewed from **Figure** **16**, the recent report from NREL,^41^ the potential to utilize continuous technologies to close the gap between laboratory cells and modules are greatly needed, and this is the purpose to develop science and technology to indicate possibility to realize optimization and precise control of non‐stoichiometric Cu–III–VI_2_ compound in the future manufacturing.

## 7 Conclusions

Materials design, with change in chemical compositions, has beendescribed, and some of the most important academic solar cell simulators have been compared. However, these simulators do not read a lot of files one by one and inevitably one analytic simulator is favored, especially for the non‐stoichiometric PV materials, based on defect concentration of the CIGS alloy. This might be one of the first steps in developing comprehensive intelligent design (materials/devices/process) and much refined work must be undertaken to further fulfill the need of PV cells for large area non‐stoichiometric materials for industrial applications. A fundamental improvement on the manufacturing technology and use of the concept of intelligent design are necessary to improve the process flow and performance of CIGS photovoltaics.
